# Supplementary material for: Distinct genomic contexts predict gene presence–absence variation in different pathotypes of Magnaporthe oryzae
Source: Genetics. 2024 Jan 30;226(4):iyae012. doi: 10.1093/genetics/iyae012 (PMC10990425; doi:10.1093/genetics/iyae012)
Supplement: iyae012_Supplementary_Data [file iyae012_supplementary_data.zip › Supplemental_Material_Legends_GENETICS-2023-306678.docx]

Supplemental Figure Legends

Fig. S1. Schematic of pipeline used to generate validated PAV events in *M. oryzae* genomes. Blue boxes describe outputs from each step of the pipeline and orange boxes describe the details of each step of the pipeline. In the cartoon phylogenies, red lines indicated gene absences and black lines indicate gene presence. A. Pipeline used to identify putative PAV events and validate them. B. Pipeline used to identify PAV orthogroups using the validated PAV matrix.

Fig. S2. Phylogeny of MoO isolates used in this study. Phylogeny was generated using a multiple-sequence alignment of SCOs and fasttree (Price *et al.* 2010). Pie charts on nodes represent the fraction of bootstrap replicates that support the node. Isolates belonging to lineage 1 are colored yellow, isolates belonging to lineage 2 are colored orange, isolates belonging to lineage 3 are colored blue, and isolates belonging to lineage 4 are colored pink. Lineages were named as previously described (Gladieux *et al.* 2018b).

Fig. S3. Phylogeny of MoT isolates used in this study. Phylogeny was generated using a multiple-sequence alignment of SCOs and fasttree (Price *et al.* 2010). Pie charts on nodes represent the fraction of bootstrap replicates that support the node. Asterisks denote isolates belonging to the recently identified pandemic clonal lineage of MoT (Latorre *et al.* 2023).

Fig. S4. Distances to the nearest gene for PAV and conserved genes in MoO and MoT. A. Density plots showing the distribution of the distances to the nearest gene for conserved and PAV genes in MoO and MoT. B. Violin plot showing the distribution of the distances to the nearest gene for conserved and PAV genes in MoO and MoT. C. Percentages and proportions of PAV and conserved genes that are within 1000bp of another gene in MoO and MoT. Dashed lines in density plots represent the median values for all genes in both pathotypes. Rectangles within violin plots represent interquartile ranges, dark lines represent medians, and dots represent the means with outliers removed. Statistics and statistical comparisons for data shown are listed in File S7, File S8, File S9, and File S10.

Fig. S5. Window-based density plots showing transposable element (TE) and gene density within genomic regions of the MoO and MoT genomes. The flanking regions of these regions are also shown. Gene- and TE-containing regions represent the subet of all deletions that overlapped at least 50% with a gene or TE sequence, respectively. Large indels were shuffled throughout the genome 100 times to generate the data for random regions in the plots.

Fig. S6. Violin plots of additional features of PAV and conserved genes. Violin plots showing the distributions of A. average flanking GC content, B. normalized H3K36me3 histone mark ChIP-Seq signal, C. noramlized H3K27ac histone mark ChIP-Seq signal, D. average % methylation of cytosines, and E. normalized extrachromosomal DNA (eccDNA) sequencing signal for PAV and conserved genes in MoO and MoT. In panels B, C, D, and E, MoT genes were not included as this data is not available for MoT. Rectangles within violin plots represent interquartile ranges, dark lines represent medians, and dots represent the means with outliers removed. Statistics describing distributions and statistical comparisons between these statistics are listed in File S11 and File S12.

Fig. S7. Comparison of various functional annotations of PAV and conserved genes. Comparison of percentages and ratios of PAV and conserved genes annotated as A. having a signal peptide, B. having a transmembrane (TM) domain, C. being a predicted effector, D. having a GO (gene ontology) annotation, and E. having a protein family (PFAM) domain annotation for MoO and MoT genes. Counts for each category and stastical comparisons of these counts are listed in File S8 and File S10.

Fig. S8. Violin plots showing the distributions of various features of MoO and MoT large indels. Violin plots showing the distributions of A. GC content, B. normalized in culture RNAseq signal, C. normalized in planta RNAseq signal, D. normalized H3K27ac histone mark ChIP-Seq signal, E. normalized H3K27me3 histone mark ChIP-Seq signal, F. normalized eccDNA sequencing signal, G. normalized H3K36me3 histone mark ChIP-Seq signal, and H. average % methylation of cytosines for large indels in MoO and MoT, as compared to baseline. In panels D-H MoT indels were not included as this data is not available for MoT. Genomic baseline values were generated by shuffling the deletions throughout the portions of the genome that were not deleted in any isolate. Rectangles within the violin plots represent interquartile ranges, dark lines represent medians, and dots represent the means with outliers removed. Statistics describing distributions and statistical comparisons between these statistics are listed in File S13.

Fig. S9. Confusion matrices showing average counts for each classification outcome for the full MoO random forest classifier, reduced MoO random forest classifier and MoT random forest classifier. A. Confusion matrix showing average counts for each classification outcome of the MoO random forest classifier when tested on MoO genes that it was not trained on. B. Confusion matrix showing average counts for each classification outcome of the MoT random forest classifier when tested on MoT genes that it was not trained on. C. Confusion matrix showing average counts for each classification outcome of the MoO random forest classifier trained on a subset of features (reduced MoO model) when tested on MoO genes that it was not trained on. D. Confusion matrix showing average counts for each classification outcome of the MoT random forest classifier when tested on MoO genes. E. Confusion matrix showing average counts for each classification outcome of the MoO random forest classifier trained on a subset of features (reduced MoO model) when tested on MoT genes.

Fig. S10. Correlation coefficients for variables included in the MoO random forest classifier. Heat map representing A. Phi coefficient between binary variables, B. Spearman rank correlation coefficient between continuous variables, and C. Point-Biserial correlation coefficient between continuous and binary variables.

Fig. S11. Dependence matrix of variables included in the MoO random forest classifier. Here, a model was trained to predict each variable used in our MoO random forest classifier using the remaining variables. A. Heatmap representing the F1 statistic of each model when trained to predict categorical variables and decrease in F1 when predictive variables were permuted in the testing data. B. Heatmap representing the R^2^ statistic of each model when trained to predict categorical variables and decrease in R^2^ when predictive variables were permuted in the testing data. Greater decreases in F1 and R^2^ statistics indicate greater dependences between variables, meaning that the predictive information encoded by these variables is most likely to already be present in other variables.

Fig. S12. Confusion matrices showing average percentages for each classification outcome for the MoT random forest classifier and the MoO random forest classifier trained on a subset of features (reduced MoO model). A. Confusion matrix showing average percentages for each classification outcome of the MoT random forest classifier when tested on MoT genes that it was not trained on. B. Confusion matrix showing average percentages for each classification outcome of the MoO random forest classifier trained on a subset of features (reduced MoO model) when tested on MoO genes that it was not trained on.

Table S1. Table of genome assemblies used in this study. Columns represent the name of the *M. oryzae* isolate, the name of the genome assembly, the host that it infects, the acronym for the pathotype, the number of scaffolds in the assembly, the number of nucleotides in the assembly, the N50 of the assembly, the L50 of the assembly and the BUSCO completeness of the assembly.

File S1: List of accessions for MoO Illumina sequencing data.

File S2: List of accessions for MoT Illumina sequencing data.

File S3: List of accessions for MoO RNAseq data.

File S4: List of large indels called using MoO Illumina sequencing data.

File S5: List of large indels called using MoT Illumina sequencing data.

File S6: Table showing the mean, median, standard deviation, 25^th^ percentile and 75^th^ percentile for the distributions of indel lengths in MoO and MoT. The p-value shown is a two-tailed p-value resulting from a permutation test for the difference in median indel length for each pathotype with 1,000 permutations.

File S7: Table showing the mean, median, standard deviation, 25^th^ percentile and 75^th^ percentile for the distributions of upstream and downstream distances to nearest PAV gene, TE, and gene for MoO and MoT. The p-values shown are two-tailed p-values resulting from permutation tests for the differences in the medians between pathotypes for PAV and conserved genes with 1,000 permutations.

File S8: Table showing the number of PAV and conserved genes that are near PAV genes, near TEs, near genes, have a TM domain, have a signal peptide, are predicted effectors, have a GO annotation, and have a PFAM domain annotation for MoO and MoT. The p-values shown were the results of Chi-squared tests used to test for indepedence between the pathotype and each feature for each PAV/conserved gene label.

File S9: Table showing the mean, median, standard deviation, 25^th^ percentile and 75^th^ percentile for the distributions of upstream and downstream distances to nearest PAV gene, TE, and gene for MoO and MoT. The p-values shown are two-tailed p-values resulting from permutation tests for the differences in the medians between PAV and conserved genes for each pathotype with 1,000 permutations.

File S10: Table showing the number of PAV and conserved genes that are near PAV genes, near TEs, near genes, have a TM domain, have a signal peptide, are predicted effectors, have a GO annotation, and have a PFAM domain annotation for MoO and MoT. The p-values shown were the results of Chi-squared tests used to test for indepedence between the PAV/conserved gene label and each feature for each pathotype.

File S11: Table showing the mean, median, standard deviation, 25^th^ percentile and 75^th^ percentile for the distributions of various continuous variables that describe PAV and conserved genes in MoO and MoT. The p-values shown are two-tailed p-values resulting from permutation tests for the differences in each statistic between PAV and conserved genes for each pathotype with 1,000 permutations.

File S12: Table showing the mean, median, standard deviation, 25^th^ percentile and 75^th^ percentile for the distributions of various continuous variables that describe PAV and conserved genes in MoO and MoT. The p-values shown are two-tailed p-values resulting from permutation tests for the differences in each statistic between pathotypes for PAV and conserved genes with 1,000 permutations.

File S13: Table showing the mean, median, standard deviation, 25^th^ percentile and 75^th^ percentile for the distributions of various continuous variables that describe large indels and baseline genomic regions in MoO and MoT. The p-values shown are two-tailed p-values resulting from permutation tests for the differences in each statistic between deletions and baseline for each pathotype with 1,000 permutations.
