# Supplementary figures and images for: Distinct genomic contexts predict gene presence–absence variation in different pathotypes of Magnaporthe oryzae
Source: Genetics. 2024 Jan 30;226(4):iyae012. doi: 10.1093/genetics/iyae012 (PMC10990425; doi:10.1093/genetics/iyae012)

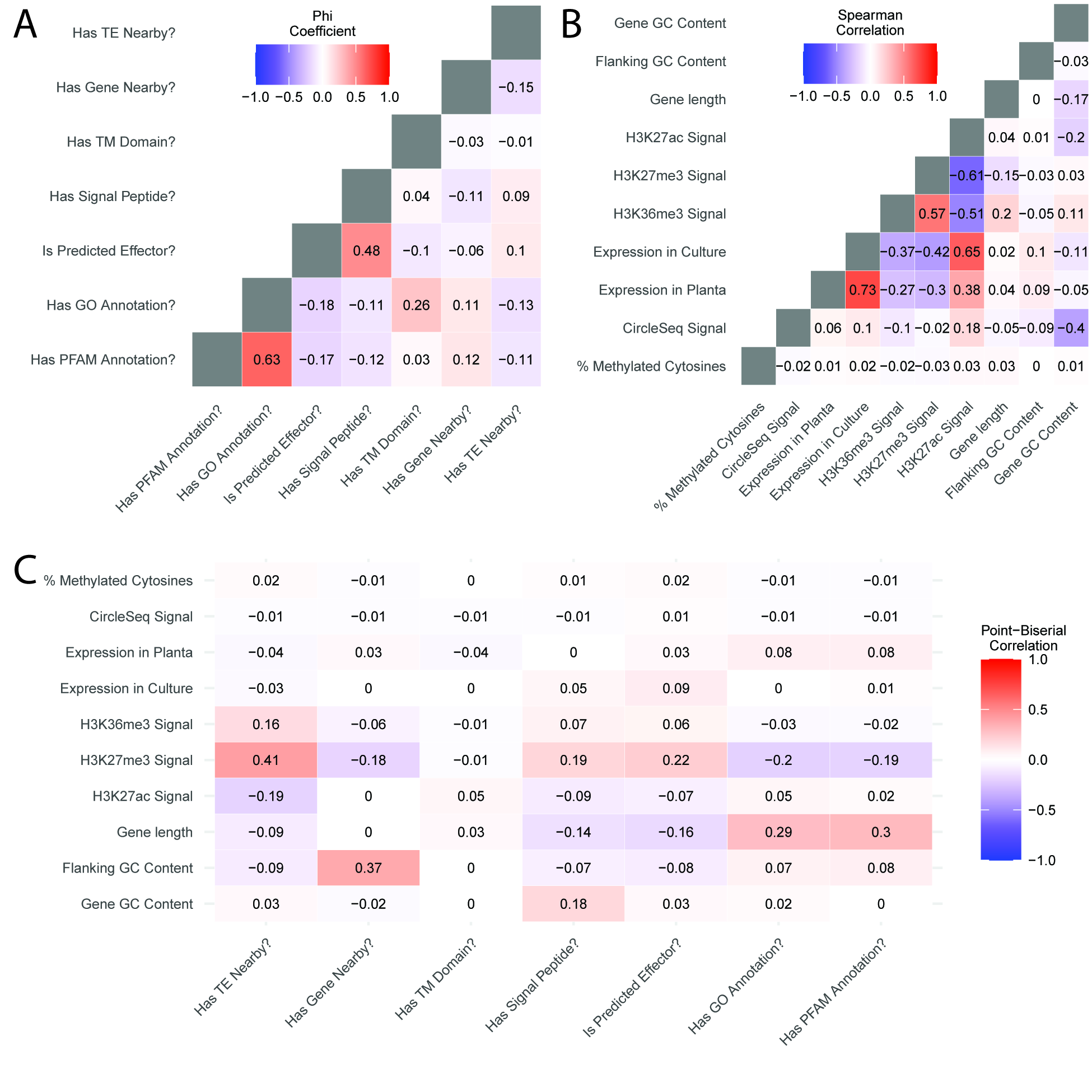

Supplement: iyae012_Supplementary_Data [file iyae012_supplementary_data.zip › Fig S10.tif]

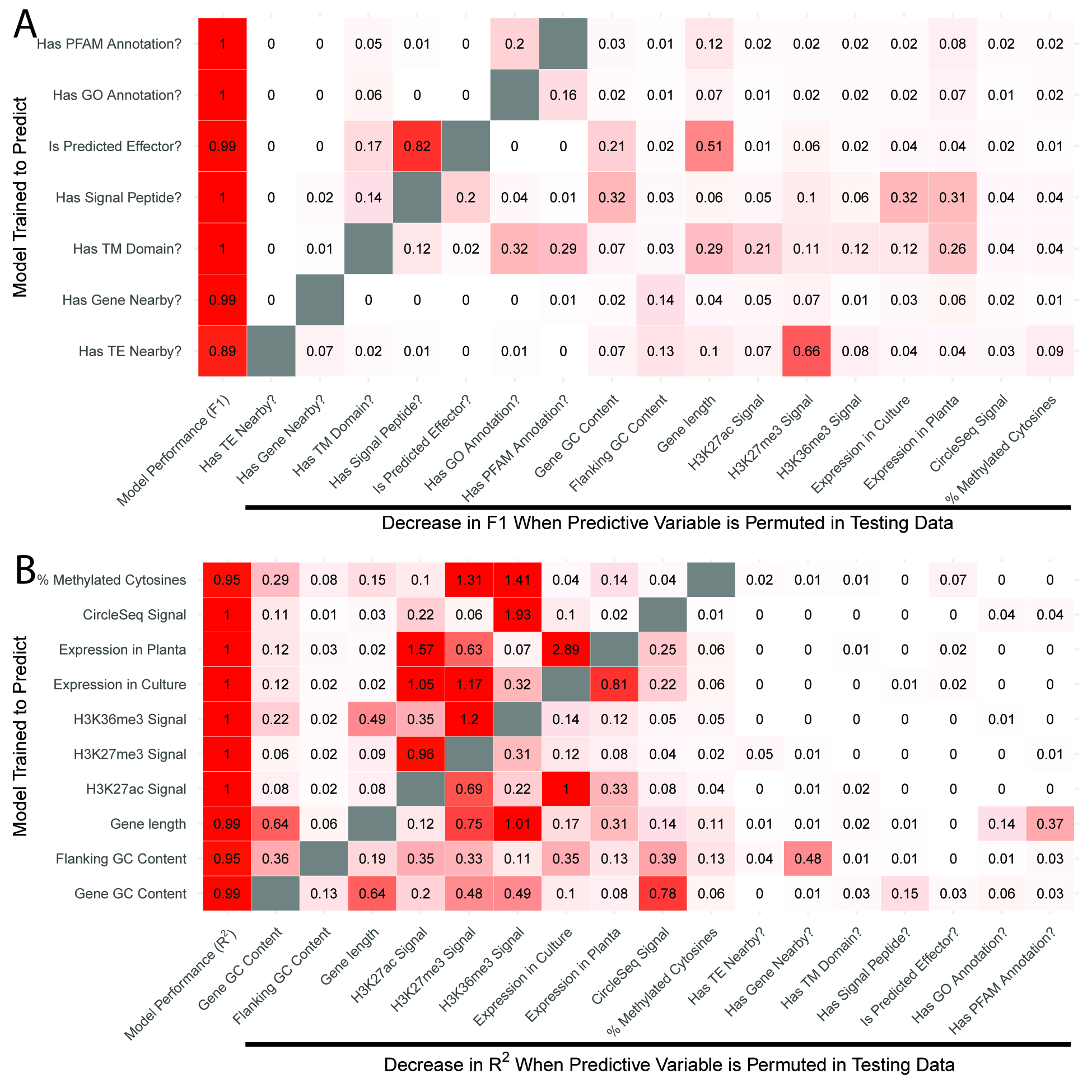

Supplement: iyae012_Supplementary_Data [file iyae012_supplementary_data.zip › Fig S11.tif]

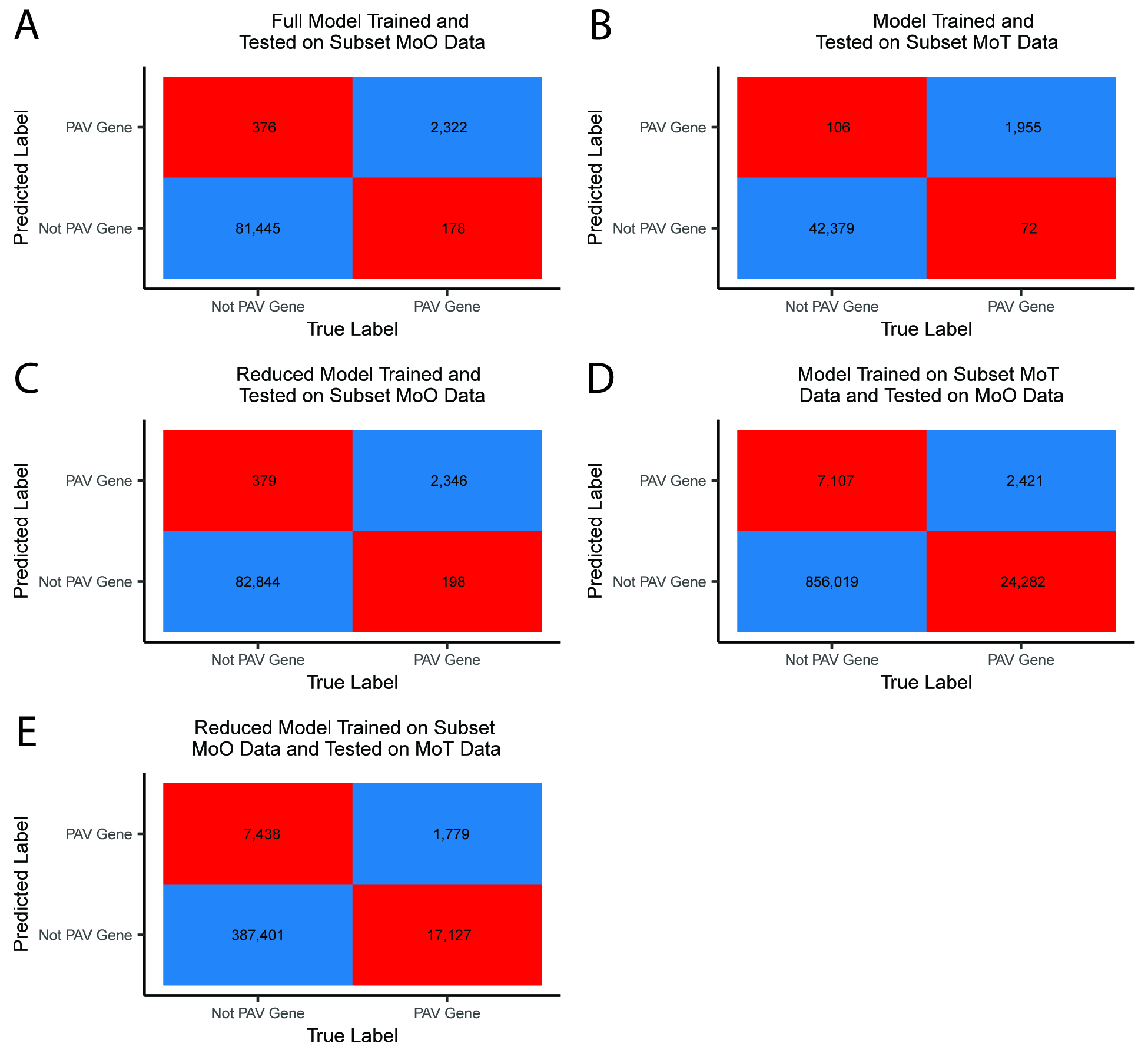

Supplement: iyae012_Supplementary_Data [file iyae012_supplementary_data.zip › Fig S9.tif]

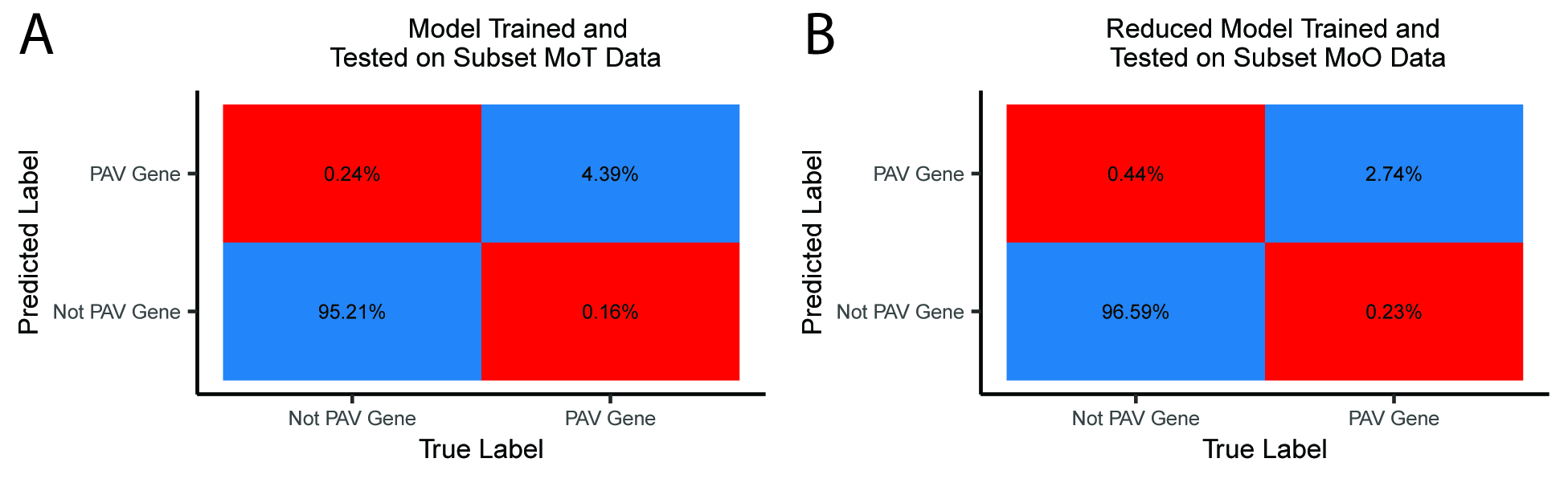

Supplement: iyae012_Supplementary_Data [file iyae012_supplementary_data.zip › Fig_S12_GENETICS-2023-306678.tif]

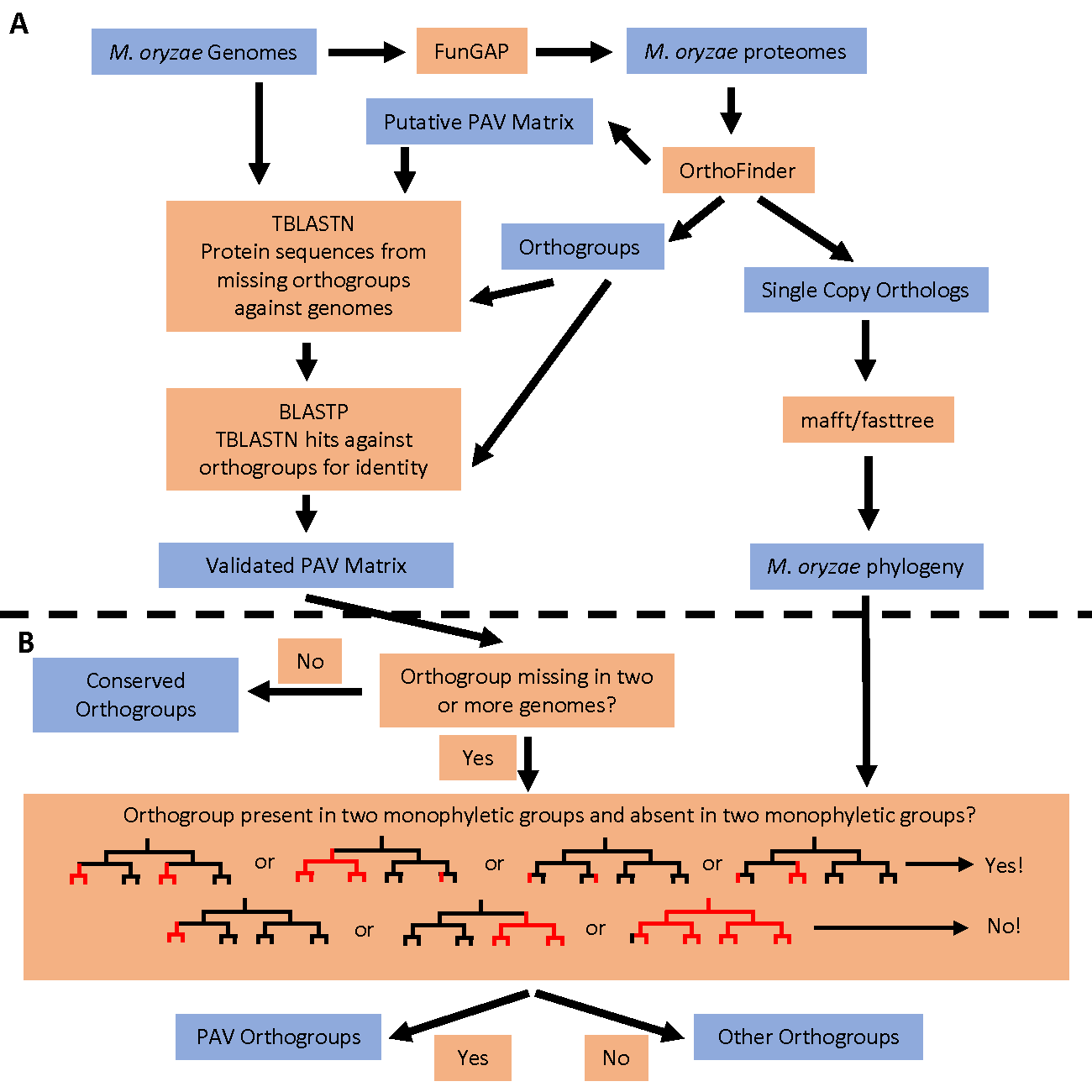

Supplement: iyae012_Supplementary_Data [file iyae012_supplementary_data.zip › Fig_S1_GENETICS-2023-306678.tif]

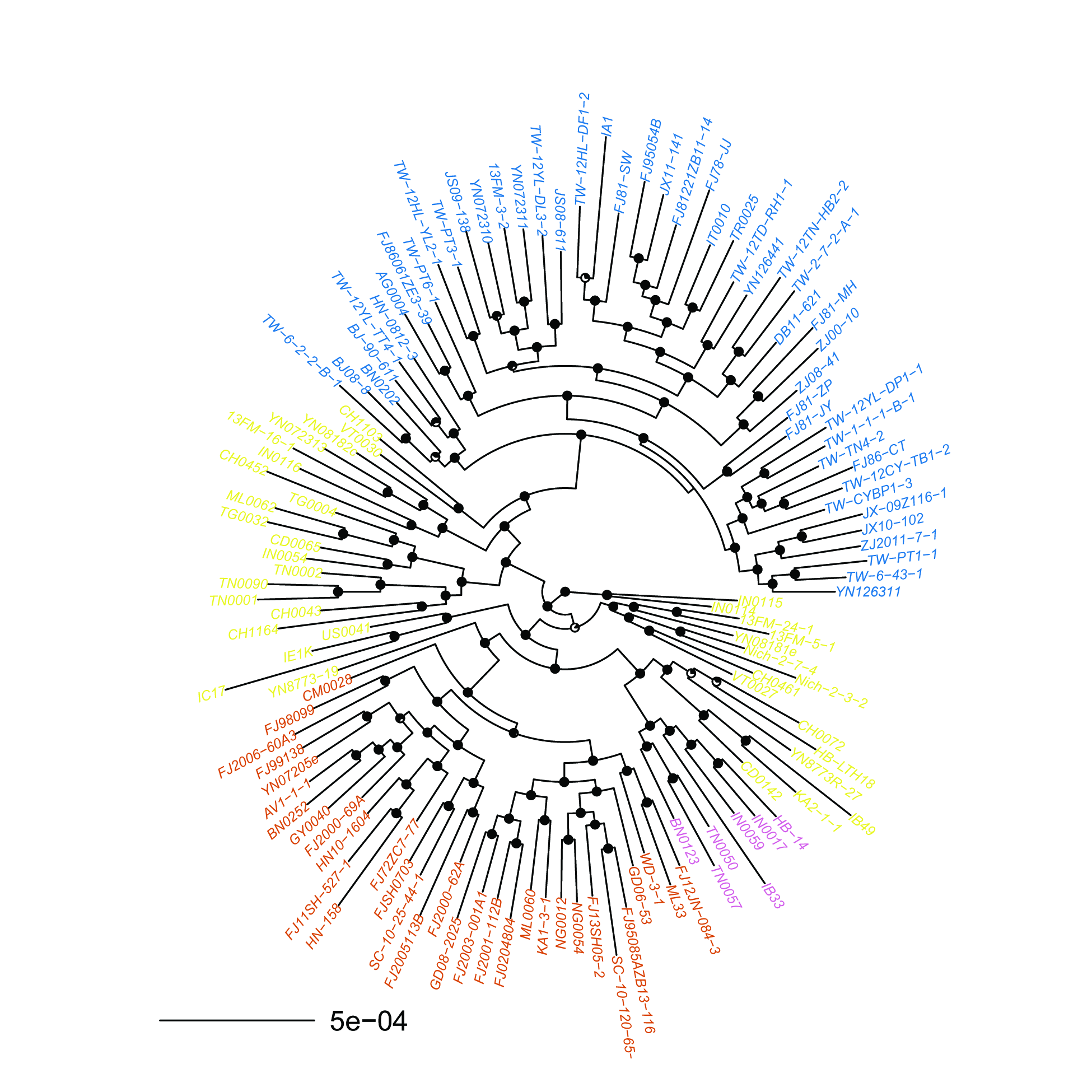

Supplement: iyae012_Supplementary_Data [file iyae012_supplementary_data.zip › Fig_S2_GENETICS-2023-306678.tif]

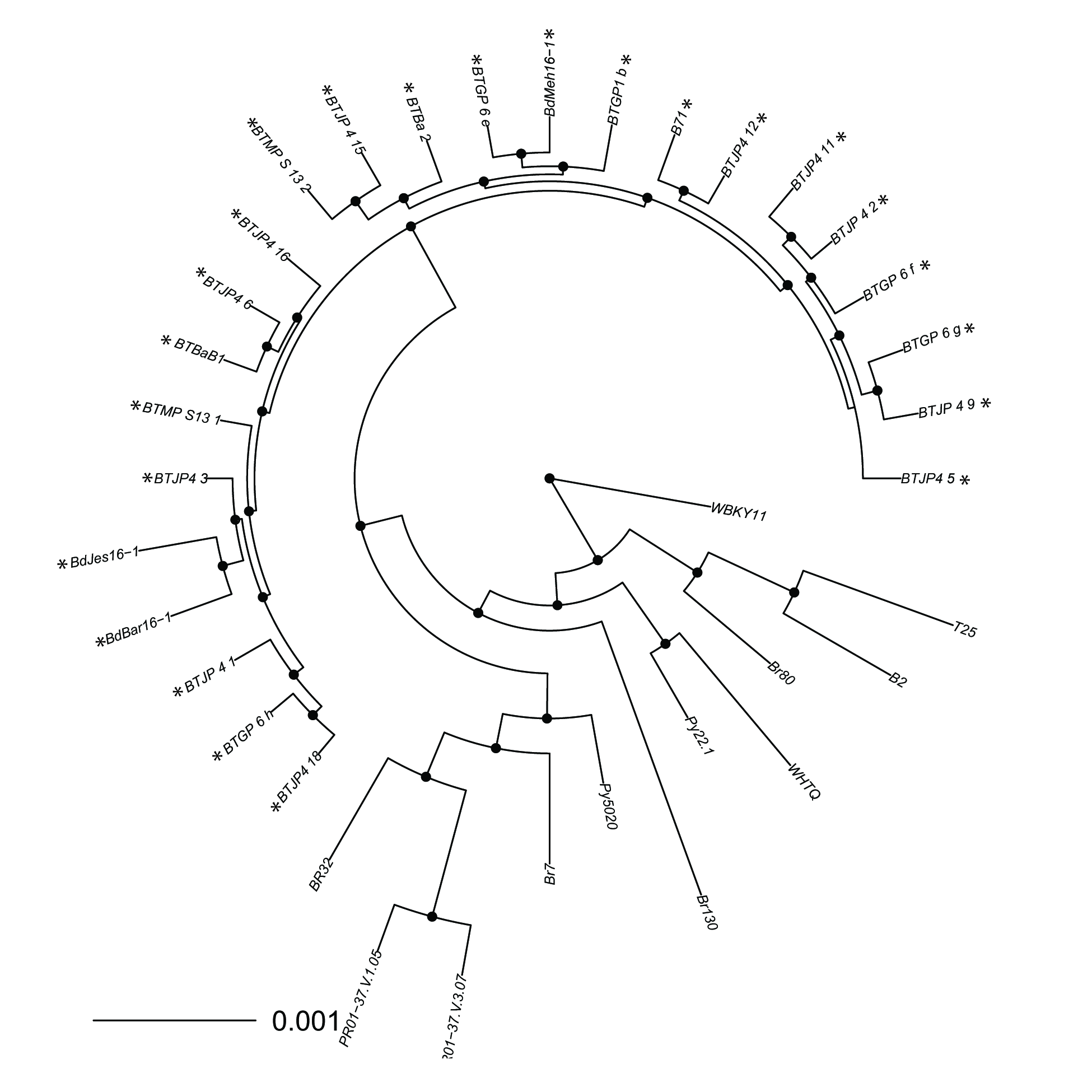

Supplement: iyae012_Supplementary_Data [file iyae012_supplementary_data.zip › Fig_S3_GENETICS-2023-306678.tif]

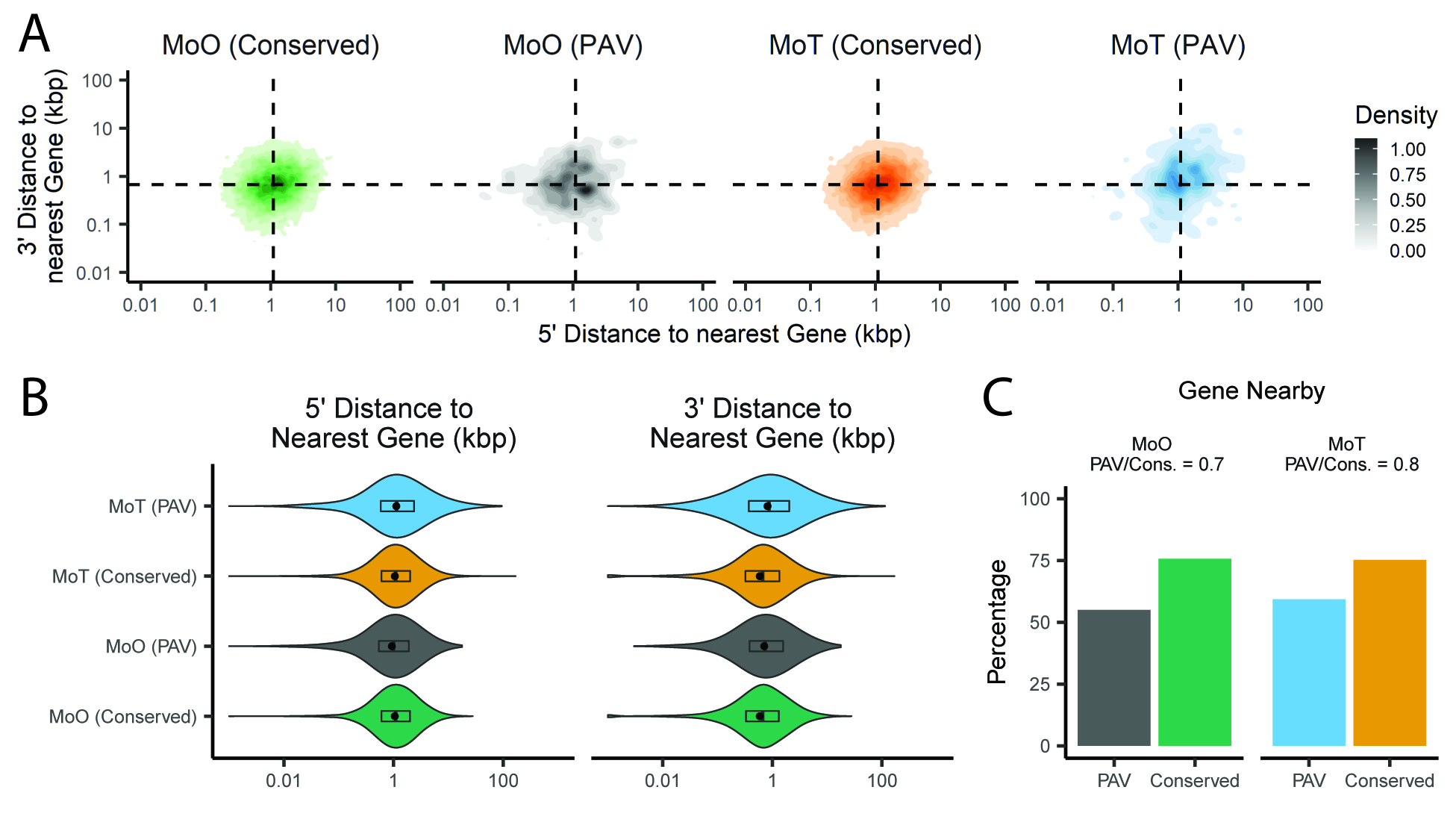

Supplement: iyae012_Supplementary_Data [file iyae012_supplementary_data.zip › Fig_S4_GENETICS-2023-306678.tif]

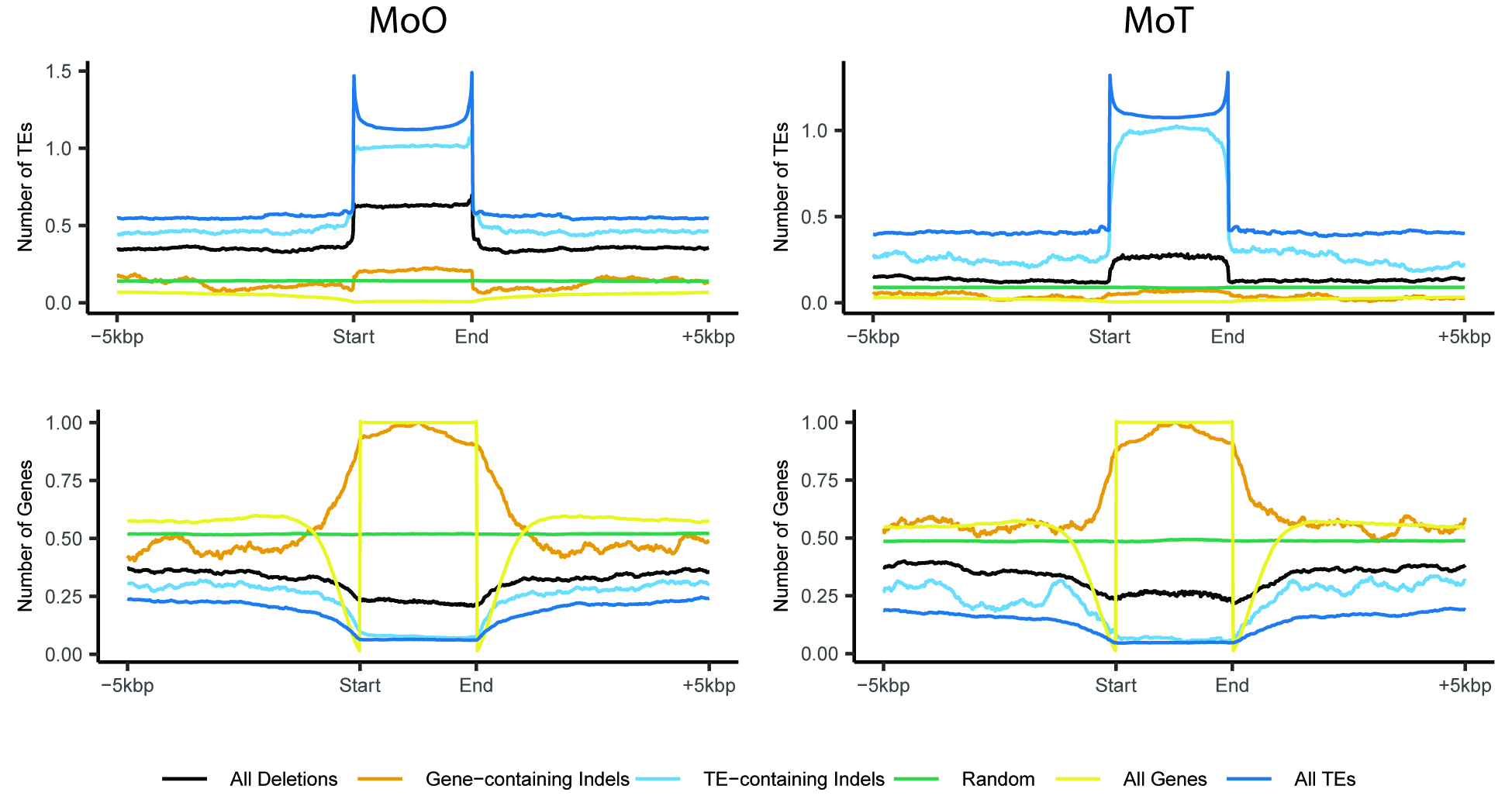

Supplement: iyae012_Supplementary_Data [file iyae012_supplementary_data.zip › Fig_S5_GENETICS-2023-306678.tif]

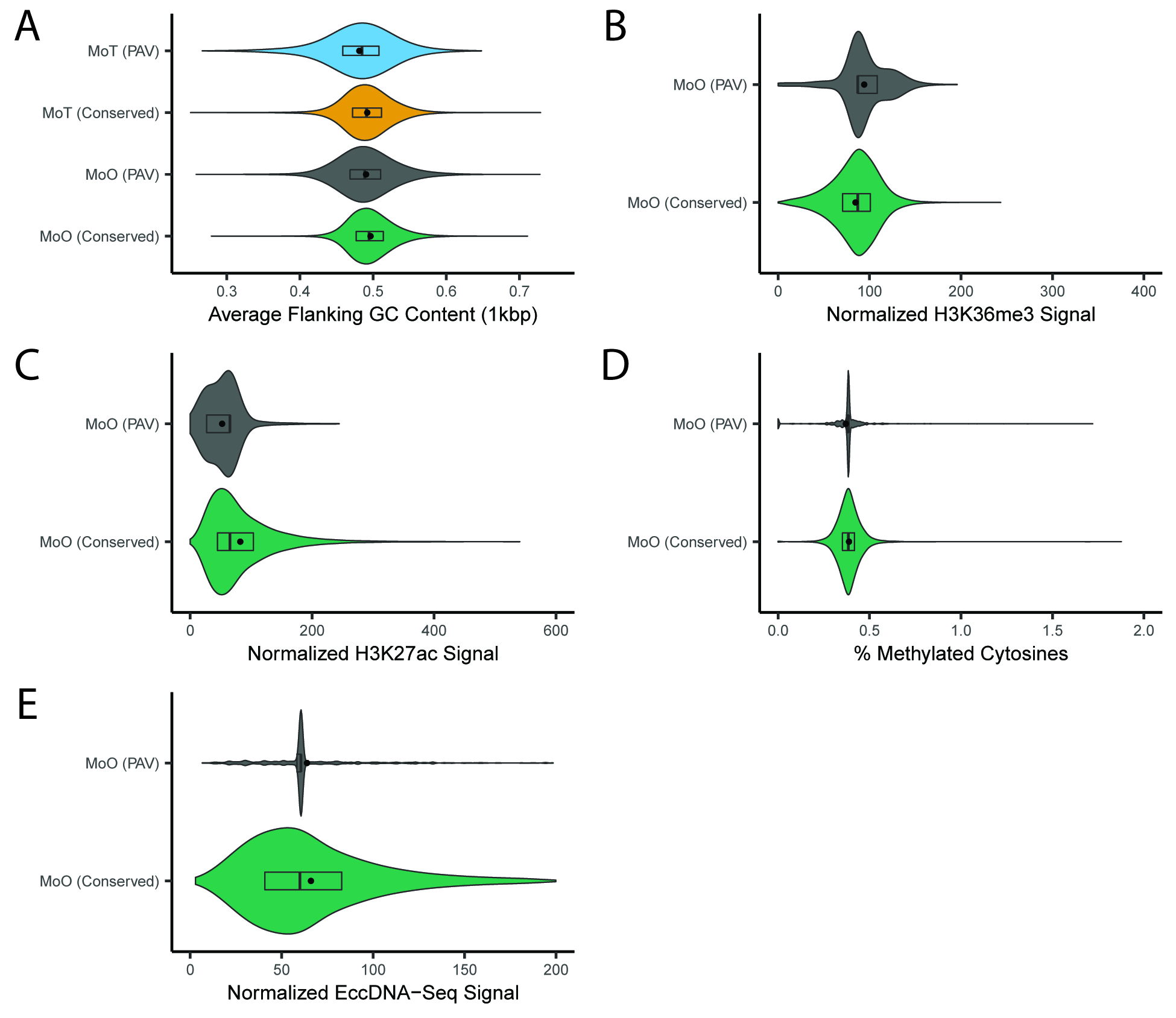

Supplement: iyae012_Supplementary_Data [file iyae012_supplementary_data.zip › Fig_S6_GENETICS-2023-306678.tif]

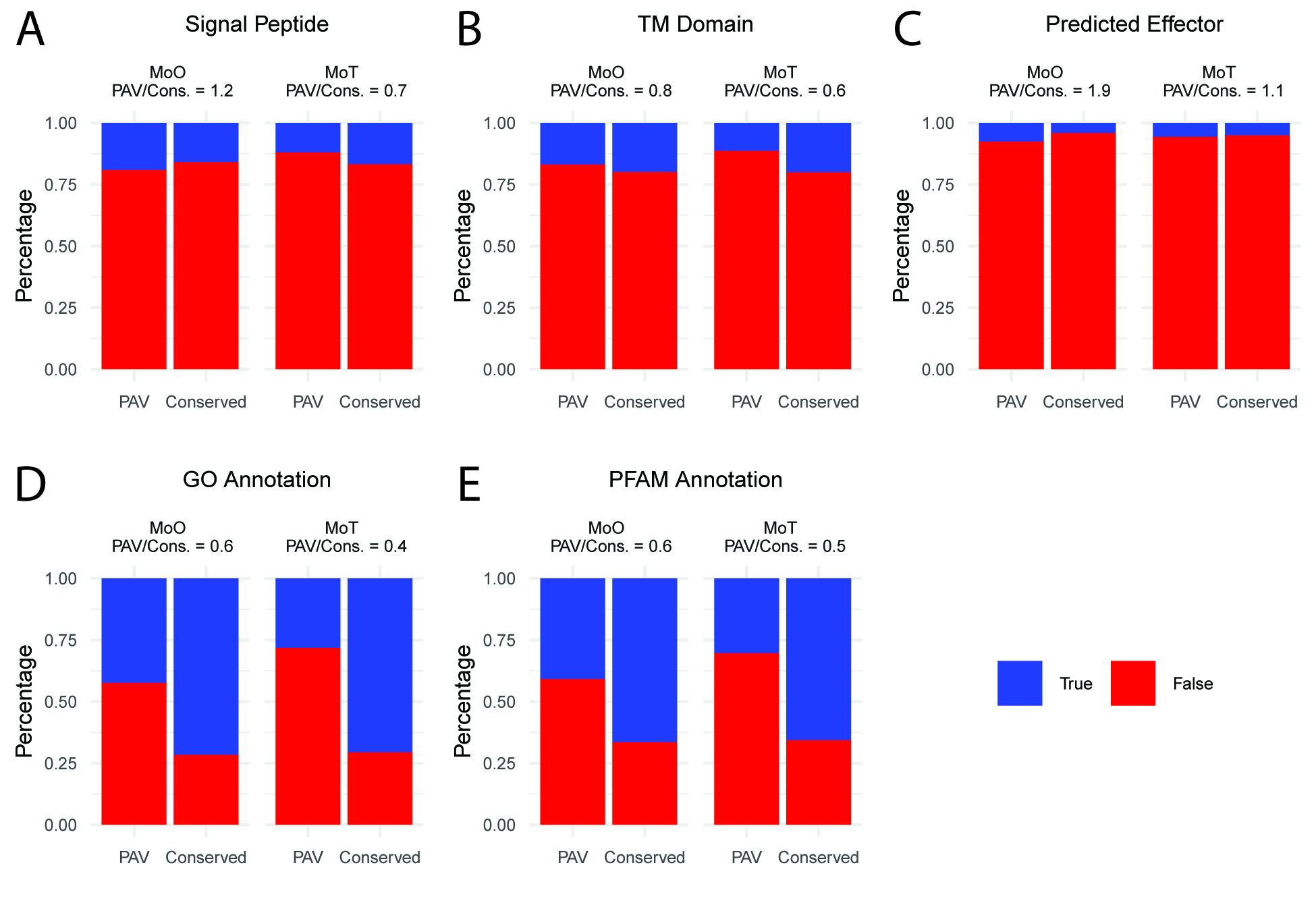

Supplement: iyae012_Supplementary_Data [file iyae012_supplementary_data.zip › Fig_S7_GENETICS-2023-306678.tif]

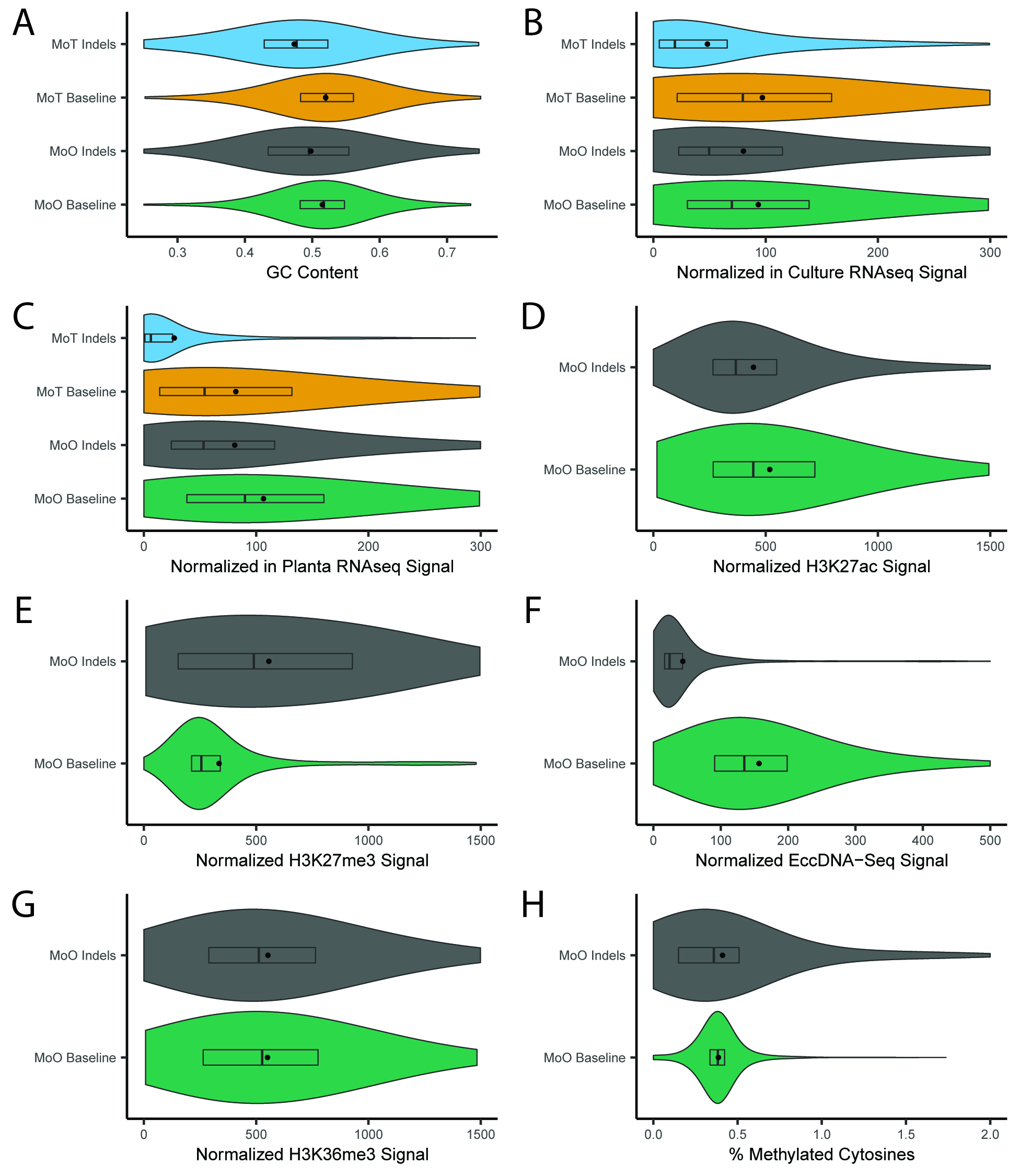

Supplement: iyae012_Supplementary_Data [file iyae012_supplementary_data.zip › Fig_S8_GENETICS-2023-306678.tif]
